# Supplementary material for: Personalized coronary and myocardial blood flow models incorporating CT perfusion imaging and synthetic vascular trees
Source: Npj Imaging. 2024 May 1;2:9. doi: 10.1038/s44303-024-00014-6 (PMC11062925; doi:10.1038/s44303-024-00014-6)
Supplement: Supplementary file 1 — Supplementary Information [file 44303_2024_14_MOESM1_ESM.pdf]

Supplementary Information: Personalized coronary and myocardial blood flow models incorporating CT perfusion imaging and synthetic vascular trees

Karthik Menon [1, 2], Muhammed Owais Khan [3], Zachary A. Sexton [4], Jakob Richter [1], Patricia K. Nguyen[5,6], Sachin B. Malik[5], Jack Boyd[7], Koen Nieman [6,8], Alison L. Marsden [1, 2, 4]

[1] *Department of Pediatrics (Cardiology), Stanford School of Medicine, Stanford, CA, USA*
[2] *Institute for Computational and Mathematical Engineering, Stanford University, Stanford, CA, USA*
[3] *Department of Electrical, Computer, and Biomedical Engineering, Toronto Metropolitan University, Toronto, ON, Canada*
[4] *Department of Bioengineering, Stanford University, Stanford, CA, USA*
[5] *VA Palo Alto Healthcare System, Palo Alto, CA, USA*
[6] *Division of Cardiovascular Medicine, Stanford School of Medicine, Stanford, CA, USA*
[7] *Department of Cardiothoracic Surgery, Stanford School of Medicine, Stanford, CA, USA*
[8] *Department of Radiology, Stanford School of Medicine, Stanford, CA, USA*

Email for correspondence: amarsden@stanford.edu

S1 . The effect of segmentation reproducibility

This section provides a brief demonstration of the robustness of the MPI_{CT}-informed modeling framework to variability in the image-based segmentation of vascular anatomical models. For this demonstration, we used the model for patient 2 as an example. This choice was motivated by computational cost, i.e. the simulations for this anatomical model were faster than those for the other patients.

The anatomical model for patient 2 was segmented from coronary computed tomography angiography (CCTA) independently from the initial segmentation performed for the data presented in the main text of this paper. Using the methods described in the main text, we built the three dimensional anatomical model and computational mesh corresponding to this new segmentation. Figure S1(a) shows a comparison of lumen diameters between the new and previous models for three important coronary arteries. Subsequently, we repeated the analysis presented in the main text to estimate coronary artery hemodynamics using Murray's law-based flow distributions and MPI_{CT}-informed flow distributions using this updated anatomy. This was performed only for models without synthetic vascular trees for simplicity. Figures S1(b) and S1(c) show comparisons between simulated and measured coronary flow fractions and myocardial blood flow (MBF) fractions for models models based on Murray's law and MPI_{CT}. As expected, the model personalization framework performed well on this new segmentation. The average error in simulated coronary flows and MBF fractions was 11.43% and 0.80% respectively. However, for models informed by Murray's law, these errors were 60.20% and 13.04%, respectively. Importantly, the new segmentation significantly affected the predicted fractional flow reserve (FFR) in the models informed by Murray's law. A comparison of FFR between the previous segmentation and new segmentation is shown in figure S1(d). In comparison, the predicted FFR was robust to segmentation variability for the models informed by MPI_{CT}, as shown in figure S1(e). This therefore suggests an added utility of this framework in the context of robustness and reproducibility.

S2 . The effect of myocardial perfusion territories

We also performed a brief investigation of the robustness of the model personalization framework to a different choice of myocardial perfusion territories corresponding to each coronary artery. For the data presented in the main text, the myocardial perfusion territory for each coronary artery was computed based on the distance of each point in the myocardium to the outlets of the coronary arteries. This is similar to other related studies in the literature [1, 4]. Another option is to compute these territories based on the closest coronary artery to each point in the myocardium, i.e. using the entire length of the coronary artery rather than just the outlet [5, 8]. We analyzed the performance of the model personalization framework using the latter definition of myocardial perfusion territories

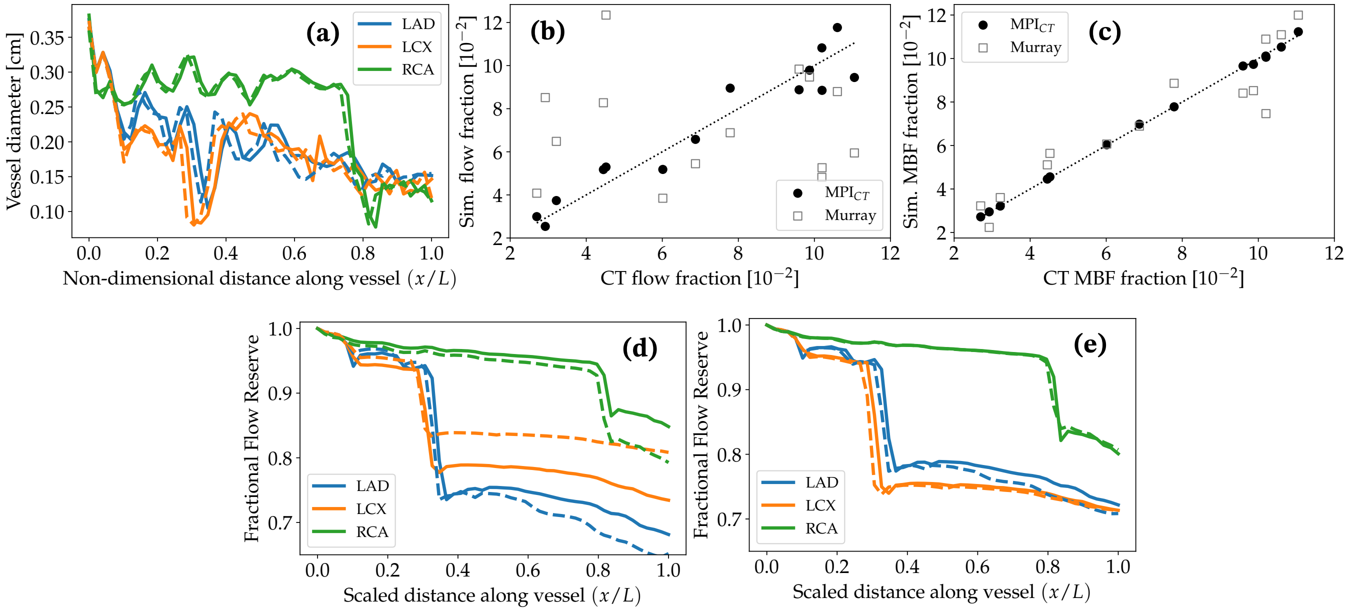

Figure S1: *(a) Comparison of lumen diameters in the previous anatomical model from the main text (solid lines) and re-segmented anatomical model (dashed lines). (b) Comparison of simulated versus measured flow fractions for simulations using boundary conditions informed by MPI$_{CT}$ and Murray's law. (c) Comparison of simulated versus measured MBF fractions for simulations using boundary conditions informed by MPI$_{CT}$ and Murray's law. (d) Comparison of simulated FFR using boundary conditions based on Murray's law between the previous (solid lines) and re-segmented (dashed lines) anatomical model. (e) Comparison of simulated FFR using boundary conditions based on MPI$_{CT}$ between the previous (solid lines) and re-segmented (dashed lines) anatomical model. LAD: Left anterior descending artery; LCX: Left circumflex artery; RCA: Right coronary artery.*

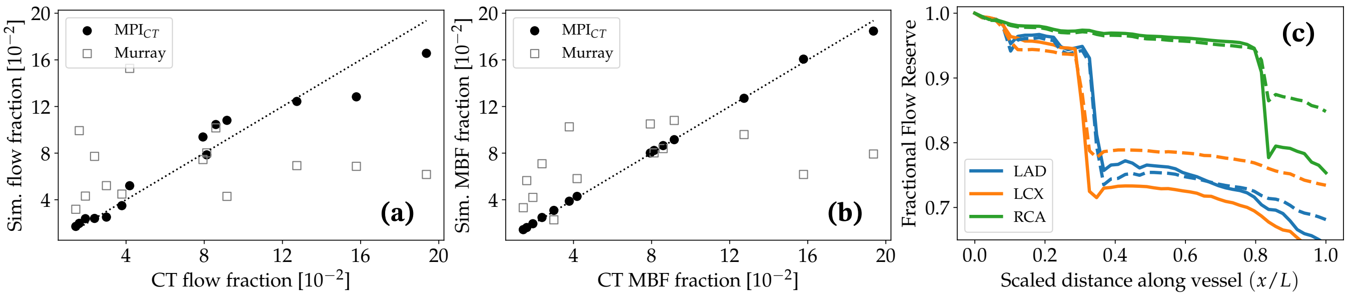

Figure S2: *(a) Comparison of simulated versus measured flow fractions for simulations using boundary conditions informed by MPI$_{CT}$ and Murray's law. (b) Comparison of simulated versus measured MBF fractions for simulations using boundary conditions informed by MPI$_{CT}$ and Murray's law. (c) Comparison of simulated FFR using boundary conditions based on MPI$_{CT}$ (solid lines) and Murray's law (dashed lines). LAD: Left anterior descending artery; LCX: Left circumflex artery; RCA: Right coronary artery.*

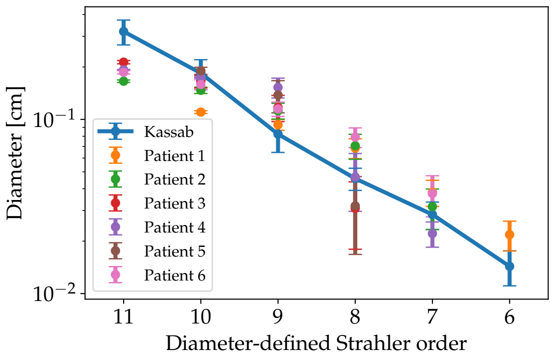

Figure S3: *Diameter-defined Strahler ordering of vessel diameters in the synthetic vascular trees compared with measured morphometric data from [3].*

using the model for patient 2 as an example (for computational simplicity, as discussed above). Figures S2(a) and S2(b) compare the simulated coronary flow and MBF fractions with measurements from MPI_{CT}. As expected, the models informed by MPI_{CT} were able to recapilate the target flow fractions, with an average error of 15.18% in coronary flow fractions and 1.71% in MBF fractions. In comparison, the models based on Murray's law had errors of 114.05% and 80.90% respectively. In addition, there was a significant difference in predicted FFR (figure S2c) between models based on Murray's law and MPI_{CT}, which followed similar trends to the data in the main text.

S3 . Comparison of synthetic vascular trees with morphometric data

The modeling pipeline used in this study employed computationally generated synthetic vascular trees to represent coronary vasculature that was too small to be accurately reconstructed from CT angiography. These synthetic trees were generated using the Constrained Constructive Optimization method [6, 7]. We assessed the physiological accuracy of these synthetic vascular trees by comparing them to the measured morphometric scaling for coronary artery trees published by Kassab *et al.* [3], as has been done in previous implementations of computationally generated synthetic trees [2]. Figure S3 shows a comparison of the synthetic vascular trees used in our modeling with the measurements of Kassab *et al.* [3] in terms of vessel diameters at various diameter-defined Strahler orders.

S4 . Clinical measurements of cardiac function

In this section we provide details of the clinical measurements and tuned model predictions for the cardiac function metrics discussed in the Results of the main text.

Patient	Measured BP	Computed BP	Measured SV	Computed SV	Measured LVEF	Computed LVEF
**1**	132/69	133/71	66.3	58.9	0.68	0.64
**2**	137/73	141/75	40.4	35.0	0.65	0.60
**3**	135/80	142/85	67.2	68.7	0.60	0.58
**4**	156/72	164/76	58.4	57.6	0.60	0.57
**5**	109/75	119/78	80.4	81.95	0.60	0.59
**6**	162/65	173/67	52.1	50.44	0.63	0.58

Table S1: *Comparison of measured and computed metrics of cardiac function for each patient. BP: Systolic/diastolic blood pressure (mmHg); SV: Stroke volume (mL); LVEF: LV ejection fraction.*

References

[1] S. Di Gregorio, M. Fedele, G. Pontone, A. F. Corno, P. Zunino, C. Vergara, and A. Quarteroni. A computational model applied to myocardial perfusion in the human heart: From large coronaries to microvasculature. *Journal of Computational Physics*, 424, 1 2021.

[2] C. Jaquet, L. Najman, H. Talbot, L. Grady, M. Schaap, B. Spain, H. J. Kim, I. Vignon-Clementel, and C. A. Taylor. Generation of Patient-Specific Cardiac Vascular Networks: A Hybrid Image-Based and Synthetic Geometric Model. *IEEE Transactions on Biomedical Engineering*, 66(4):946–955, 4 2019.

[3] G. S. Kassab, C. A. Rider, N. J. Tang, and Y. C. Fung. Morphometry of pig coronary arterial trees. *American Journal of Physiology - Heart and Circulatory Physiology*, 265(1):350–365, 1993.

[4] L. Papamanolis, H. J. Kim, C. Jaquet, M. Sinclair, M. Schaap, I. Danad, P. van Diemen, P. Knaapen, L. Najman, H. Talbot, C. A. Taylor, and I. Vignon-Clementel. Myocardial Perfusion Simulation for Coronary Artery Disease: A Coupled Patient-Specific Multiscale Model. *Annals of Biomedical Engineering*, 49(5):1432–1447, 2021.

[5] J. T. Schrauwen, A. Coenen, A. Kurata, J. J. Wentzel, A. F. van der Steen, K. Nieman, and F. J. Gijsen. Functional and anatomical measures for outflow boundary conditions in atherosclerotic coronary bifurcations. *Journal of Biomechanics*, 49(11):2127–2134, 2016.

[6] W. Schreiner and P. F. Buxbaum. Computer-Optimization of Vascular Trees. *IEEE Transactions on Biomedical Engineering*, 40(5):482–491, 1993.

[7] Z. A. Sexton, A. R. Hudson, J. E. Herrmann, D. J. Shiwarski, J. Pham, J. M. Szafron, S. M. Wu, M. Skylar-Scott, A. W. Feinberg, and A. Marsden. Rapid model-guided design of organ-scale synthetic vasculature for biomanufacturing. *arXiv*, 2308.07586, 2023.

[8] X. Xue, X. Liu, Z. Gao, R. Wang, L. Xu, D. Ghista, and H. Zhang. Personalized coronary blood flow model based on CT perfusion to non-invasively calculate fractional flow reserve. *Computer Methods in Applied Mechanics and Engineering*, 404:115789, 2023.
